# Plant diversity and soil legacy independently affect the plant metabolome and induced responses following herbivory

**DOI:** 10.1002/ece3.10667

**Published:** 2023-11-02

**Authors:** Christian Ristok, Nico Eisenhauer, Alexander Weinhold, Nicole M. van Dam

**Affiliations:** ^1^ German Centre for Integrative Biodiversity Research (iDiv) Halle‐Jena‐Leipzig Leipzig Germany; ^2^ Institute of Biodiversity Friedrich Schiller University Jena Jena Germany; ^3^ Leipzig University Leipzig Germany; ^4^ Leibniz Institute of Vegetable and Ornamental Crops (IGZ) Großbeeren Germany

**Keywords:** aboveground–belowground interactions, biodiversity–ecosystem function, chemical diversity, eco‐metabolomics, herbivory, metabolite profile

## Abstract

Plant and soil biodiversity can have significant effects on herbivore resistance mediated by plant metabolites. Here, we disentangled the independent effects of plant diversity and soil legacy on constitutive and herbivore‐induced plant metabolomes of three plant species in two complementary microcosm experiments. First, we grew plants in sterile soil with three different plant diversity levels. Second, single plant species were grown on soil with different plant diversity‐induced soil legacies. We infested a subset of all plants with *Spodoptera exigua* larvae, a generalist leaf‐chewing herbivore, and assessed foliar and root metabolomes. Neither plant diversity nor soil legacy had significant effects on overall foliar, root, or herbivore‐induced metabolome composition. Herbivore‐induced metabolomes, however, differed from those of control plants. We detected 139 significantly regulated metabolites by comparing plants grown in monocultures with conspecifics growing in plant or soil legacy mixtures. Moreover, plant–plant and plant–soil interactions regulated 141 metabolites in herbivore‐induced plants. Taken together, plant diversity and soil legacy independently alter the concentration and induction of plant metabolites, thus affecting the plant's defensive capability. This is a first step toward disentangling plant and soil biodiversity effects on herbivore resistance, thereby improving our understanding of the mechanisms that govern ecosystem functioning.

## Graphical abstract

We experimentally disentangled plant diversity and soil biodiversity testing for the independent effects of plant–plant interactions and plant–soil interactions on constitutive and herbivore‐induced plant metabolomes. We found evidence for the independent effects of plant diversity and soil biodiversity thus advancing our mechanistic understanding of plant–soil‐herbivore interactions.

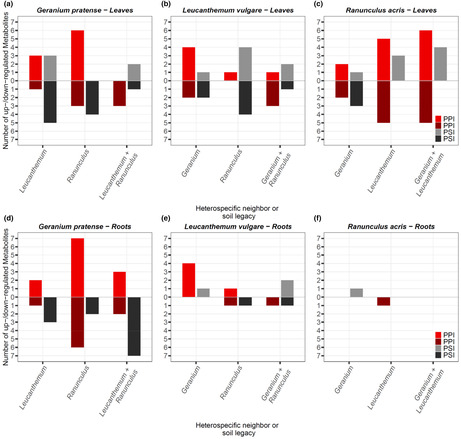

## 1 INTRODUCTION

### 1.1 Plant and soil communities are linked via the plant and influence each other

In terrestrial ecosystems, aboveground and soil communities are inseparably linked via plants (Wardle et al., 2004). Such aboveground–belowground linkages determine plant diversity effects on ecosystem functioning (Eisenhauer, 2012). Plant species often harbor unique rhizosphere communities and even influence the surrounding community composition of root‐associated organisms through species‐specific and context‐dependent organic matter inputs (Bezemer et al., 2010; van der Putten et al., 2013). Plant species‐specific root exudates, for instance, can both increase and reduce soil pathogens of neighboring heterospecific plants (Steinauer et al., 2016; van de Voorde et al., 2011). These plant‐induced changes in soil biota can persist over time and result in soil legacy effects (Kardol et al., 2007). Similarly, soil biota, especially root parasites, pathogens, and herbivores as well as mutualistic symbionts can influence plant community structure and functioning via soil feedback effects (Van Der Heijden et al., 2008; van der Putten et al., 2013; Wardle et al., 2004). Soil feedback effects are considered positive when the performance or fitness of individual plants is increased and are considered negative when plant performance or fitness is reduced (Ehrenfeld et al., 2005; Kulmatiski et al., 2008; van der Putten et al., 2013). Root parasites, pathogens, and herbivores generally induce a negative soil feedback effect, for instance, by directly removing or damaging root tissues and thus reducing root uptake capabilities. Mutualistic organisms, on the other hand, induce a positive soil feedback effect on plant growth by improving soil nutrient uptake (Bardgett & van der Putten, 2014; Wardle et al., 2004), and protection against antagonists (Latz et al., 2012). The magnitude and direction of those soil feedback effects, however, is not equal for all plant species and community contexts (Cortois et al., 2016). In turn, each plant species has a specific herbivore community which can affect soil communities via herbivory, either directly via frass or indirectly via induced responses (Bardgett & Wardle, 2010). Aboveground herbivory can, for instance, positively affect soil microbial activity by inducing the release of carbon into the rhizosphere, and change arbuscular mycorrhizal colonization by reducing the carbon allocation to roots (Gehring & Whitham, 1994; Hamilton & Frank, 2001).

### 1.2 Plant diversity and soil legacy can affect the plant metabolome

Recently, research on the response of plants to plant–plant interactions and soil feedbacks has been expanded beyond the common morphological and physiological traits. Plant–plant interactions, for instance, can induce shifts in foliar metabolic profiles of multiple grassland plant species, with more than 100 metabolites changing in their concentration (Scherling et al., 2010), potentially related to competition and the production of allelopathic metabolites (Fernandez et al., 2016; Treutter, 2006). Similarly, in the presence of soil biota, plants produce species‐specific shoot and root metabolomes that differ from those of plants grown in sterile conditions (Ristok et al., 2019), potentially related to mycorrhization, the interaction with nematodes and priming (Conrath et al., 2006; Rivero et al., 2015; Wurst et al., 2010). The consideration of the plant metabolome, that is, the entirety of metabolites synthesized by a plant (Oliver et al., 1998) gave rise to a new discipline, eco‐metabolomics, which uses metabolome analyses, or metabolomics, to illuminate the chemical mechanisms underpinning ecological and environmental processes (Peñuelas & Sardans, 2009; Peters et al., 2018). Eco‐metabolomics has been employed to investigate if plants respond on a molecular level to plant community composition and soil biota diversity (Huberty et al., 2020; Ristok et al., 2019; Scherling et al., 2010). This response can entail the de‐novo synthesis of compounds, changes in metabolite concentration through regulation, and differences in overall metabolome composition (often due to a change in concentrations of a large number of metabolites).

Moreover, differences in plant species richness can affect plant metabolome composition, with the most pronounced metabolome differences observed in plants grown in monoculture compared to plants grown in high‐diverse plant communities (Ristok, Weinhold, et al., 2023). In addition, differential selection due to growing in monocultures or plant species mixtures can select for plants with distinct metabolomes (Zuppinger‐Dingley et al., 2015). Furthermore, plant–soil interactions often affect the diversity of a plant's metabolome and can exert stronger metabolomic shifts than foliar herbivory (Huberty et al., 2020). The interaction with root parasites, pathogens, and herbivores as well as mutualistic symbionts can change the concentration of primary and secondary metabolites in leaves and roots in multiple ways, for example, up‐ or downregulation of specific metabolites (van Dam & Heil, 2011; van der Putten et al., 2013). These responses are generally species‐specific, context‐dependent, and can affect subsequent biotic interactions, such as with aboveground herbivores (Bezemer & van Dam, 2005; Ristok et al., 2019; Ristok, Weinhold, et al., 2023).

### 1.3 Herbivory‐induced defenses can be altered by biotic interactions

Plants have evolved a plethora of indirect and direct chemical defenses to deal with attackers (Karban & Baldwin, 1997). Induced defenses, that is, changes in the concentration of metabolites following an attack by parasites, pathogens or herbivores, or after interactions with beneficial microbes (Ferlian et al., 2018) can affect the plant metabolome locally or systemically (Bezemer & van Dam, 2005). Both plant–plant interactions and plant–soil interactions can modulate the induction of defensive metabolites. Plant–plant interactions can affect induced defenses through plant competition, which can drive the plant to either invest resources into growth or defense (Broz et al., 2010; Fernandez et al., 2016; i.e., growth‐defense trade off; van Dam & Baldwin, 2001). In addition, volatile organic compounds can induce defensive responses immediately or prime for future attacks (Baldwin et al., 2006). Plant–soil interactions with microbes, nematodes, and mycorrhizal fungi cannot just induce defenses locally in roots, but also systemically in foliar tissues (van Dam & Heil, 2011). Interactions with either of these groups of soil biota can trigger up‐ or downregulation of specific primary metabolites, such as amino acids and sugars, or secondary metabolites, such as glucosinolates and iridoid glycosides, in aboveground and belowground plant tissues (Hol et al., 2010; Rivero et al., 2015; Wurst et al., 2010).

Taken together, both plant–plant interactions and plant–soil interactions play significant roles in modulating the plant's metabolome, thereby affecting resistance to aboveground herbivores (Ristok et al., 2019; Ristok, Weinhold, et al., 2023; van Dam & Heil, 2011). Thus far, however, not much is known about the individual impact of plant–plant interactions or plant–soil interactions within plant communities. This is likely due to the fact that plant–plant and plant–soil interactions are tightly linked in natural communities. In addition, most microcosm studies only focus on plant–soil interaction effects (see e.g., Huberty et al., 2020; Ristok et al., 2019). Here, we explicitly investigate to which extent plant–plant‐interactions (PPI) or plant–soil interactions (PSI) affect the metabolomes of three forb species in a similar microcosm setup. Both the PPI and PSI experiment covered the same range of diversity levels and plant community compositions; either as assembled plant communities grown in sterile soil (PPI) or via the inoculation of sterile substrate with conditioned field soil of communities with similar plant diversity levels (PSI). This experimental setup minimized the direct effect of soil biota in the PPI experiment through the use of sterile soil, and minimized the direct effect of heterospecific plants in the PSI experiment by planting only monospecific communities. In addition, a subset of all plants was infested with larvae of the generalist herbivore *Spodoptera exigua* to induce defense responses. We analyzed all samples using an untargeted metabolomics approach focusing on profiling plant secondary metabolites in leaves and roots, because secondary metabolites help plants cope with their (a)biotic environment and are involved in many biotic interactions (van Dam, 2009b; van Dam & van der Meijden, 2011; Whitehead et al., 2021). We defined PPI effects as changes in a plant's secondary metabolite profile due to direct effects of heterospecific plants, such as changes in resource availability or root exudation. In comparison, we defined PSI effects as changes in a plant's secondary metabolite profile due to direct interactions with soil biota, such as bacteria, fungi, pathogens, and nematodes. We hypothesized that (1) both plant diversity and soil legacy can alter the overall plant metabolome, as well as affect the regulation of specific metabolites. In addition, we hypothesized that (2) the induced metabolomic response to herbivory is differently affected by plant diversity and soil legacy.

## 2 MATERIALS AND METHODS

### 2.1 Experimental design

In summer 2017, we set up a plant–plant interaction (PPI) experiment and a plant–soil interaction (PSI) experiment with three common central European grassland forb species (*Geranium pratense* L., *Leucanthemum vulgare* (Vaill.) Lam., and *Ranunculus acris* L.). We chose these species based on their representation in the Trait‐Based Experiment of the Jena Experiment (Ebeling et al., 2014), that is, monocultures of each species, all two‐species mixtures, and the three‐species mixture were established (see below). Prior to each experiment, we germinated seedlings of each species from non‐sterilized seeds (Rieger‐Hofmann GmbH). To assure that we would use similarly developed seedlings and to account for species‐specific differences in germination, we treated the seeds as follows (based on prior germination trials; data not shown): all seeds of *Geranium pratense* were gently scarified with sandpaper, placed in a petri dish, and treated with 3 mL 1 g/L gibberellic acid for 24 h at 7°C. The same procedure was followed for *Ranunculus acris* seeds, but they were treated with 0.66 g/L gibberellic acid. No treatment was necessary for *Leucanthemum vulgare* seeds. Following the treatment, all seeds were transferred to plastic boxes half‐filled with glass beads (50 seeds per box, only one species per box). Each box was covered with a transparent lid, and seeds were watered daily with tap water. All boxes were transferred to growth chambers (CLF Plant Climatics, Percival E‐36L) with a photoperiod of 16 h light at 20°C and 8 h darkness at 12°C, and 50% relative humidity. To ensure that all seedlings reached similar sizes, seeds of *R. acris* were prepared and moved to the growth chamber 2 weeks before those of *G. pratense* and *L. vulgare* (i.e., seeds of *R. acris* were left 28 days and seeds of *G. pratense* and *L. vulgare* were left 14 days in the growth chamber).

#### 2.1.1 Plant–plant interaction experiment

We conducted the plant–plant interaction experiment in a greenhouse located at the Botanical Garden Leipzig, Germany, in May 2017. We recorded an average temperature of 22.6°C and an average relative humidity of 51.6% for the time of the experiment in the greenhouse. We used 2 L microcosms (rose pot 2.0 L; Hermann Meyer KG) filled with autoclaved (twice at 134°C for 20 min) 50:50 sand‐peat (Floradur B Pot Clay Medium; Floragard) mixture. We flushed each filled microcosms with water twice to remove pulsed nutrients and toxins prior to transplanting seedlings (Alphei & Scheu, 1993; Trevors, 1996). To allow for similar soil conditions between the plant–plant interaction experiment and the plant–soil interaction experiment (see below), we chose to use a commercial sand‐peat mixture as it was not possible to retrieve enough soil from the field site in Jena, Germany. We established the following plant diversity levels and communities: (1) monocultures of each species, (2) the three possible two‐species mixtures, and (3) the three‐species mixture (Table A1). We transplanted 12 similarly developed seedlings in each microcosm, and each plant community was replicated 10 times (total number of microcosms: 70). The relative proportion among species was equal, that is, six seedlings per species in the two‐species mixture and four seedlings per species in the three‐species mixture. In the two‐species mixture, we transplanted the species in an alternating pattern, while we randomized the position of each seedling in the three‐species mixture. All microcosms were randomly placed on tables in the greenhouse and covered with net cages to prevent unwanted herbivory. We observed no visual signs of light deficiency and thus assume that no additional variation was introduced by the net cages. We watered all microcosms three times per week and randomized the position on the tables every 7 days. We fertilized all microcosms with 250 mL Hoagland solution, that is, “half‐Hoagland solution”, after 5 weeks to counteract any loss of nutrients and ensure optimal growth.

After 7 weeks of growth, we harvested five microcosms per plant diversity level to assess the constitutive metabolome (see below). The next day, we infested two randomly selected plants per species and microcosm of the remaining microcosms with three 2nd instar *Spodoptera exigua* larvae each. We covered and closed each plant just above the soil with an organza net to ensure that the larvae could not escape. To ensure similar development of the larvae (eggs purchased from Entocare Biologische Gewasbescherming, Wageningen, the Netherlands), we maintained a laboratory colony on artificial diet (Elvira et al., 2010) in a growth chamber (25°C, 12 h light, 45% relative humidity). After 7 days of herbivory, we harvested the remaining microcosms to assess the herbivore‐induced metabolome (see below).

#### 2.1.2 Plant–soil interaction experiment

We conducted the plant–soil interaction experiment in a greenhouse located at the Botanical Garden Leipzig, Germany, in July 2017. We recorded an average temperature of 23.5°C and an average relative humidity of 58.6% for the time of the experiment in the greenhouse. We used PVC tube microcosms (height 20 cm, diameter 10 cm, bottom closed with 250 μm mesh) filled with 1.6 L inoculated substrate and watered each microcosm twice. We prepared the inoculated substrate by mixing autoclaved (twice at 134°C for 20 min) 50:50 sand‐peat (Floradur B Pot Clay Medium; Floragard) background substrate with liquid field soil inoculum 3 weeks prior to the establishment of the experiment. In June 2017 (i.e., ~7 years after the establishment of the experiment), we collected field soil from plant communities established in 2010 as part of the Trait‐Based Experiment (Ebeling et al., 2014). We collected and pooled six soil cores (2 cm × 10 cm) from each plant community accounting for within‐plot heterogeneity. We sieved each field soil through a 4 mm mesh and subsequently dissolved 100 g field soil in 1 L demineralized water. We then added the liquid soil inoculum to our autoclaved background substrate (10 mL liquid inoculum per 1 kg background substrate) and stored each mixture in closed‐lid plastic boxes at room temperature for 3 weeks. Each substrate‐inoculum mixture was thoroughly mixed three times per week and stored with an open lid for 1 h once per week. We cleaned all used instruments, that is, sieves, boxes, beakers, mixer, before and after each step with distilled water and 70% ethanol to minimize cross contamination.

We established the following inoculated substrates (hereafter, soil legacy levels): (1) monocultures of each plant species, (2) the three possible two‐species mixtures, and (3) the three‐species mixture (Table A2). Each soil legacy level represents the plot from the Trait‐Based Experiment, we sampled the soil from. We transplanted four similarly developed seedlings per microcosm. Seedlings of plant species were only planted into soil legacy levels that also contained the respective species in the field experiment. This setup resulted in 12 unique soil legacy level‐planted species combinations. Each soil legacy level‐planted species combination was replicated 10 times (total number of microcosms: 120). All microcosms were randomly placed on tables in the greenhouse and covered with net cages to prevent unwanted herbivory. We observed no visual signs of light deficiency and thus assume that no additional variation was introduced by the net cages. We watered all microcosms three times per week and randomized the position on the tables every 7 days. We fertilized all microcosms with 250 mL Hoagland solution, that is, “half‐Hoagland solution”, after 5 weeks to counteract any loss of nutrients and ensure optimal growth. After 7 weeks of growth, we harvested five microcosms per soil legacy level‐planted species combination to assess the constitutive metabolome (see below). The next day, we infested two randomly selected plants per microcosms of the remaining microcosms with three 2nd instar *Spodoptera exigua* larvae each (see above). We covered and closed each plant just above the soil with an organza net to ensure that the larvae could not escape. After 7 days of herbivory, we harvested the remaining microcosms to assess the herbivore‐induced metabolome (see below).

### 2.2 Sampling and sample processing

After 7 weeks of growth, we harvested five microcosms per plant diversity level in the PPI experiment and five microcosms per soil legacy level‐planted species combination in the PSI experiment (Tables A1 and A2). We separated the shoot and root biomass of one randomly selected plant individual per species and microcosm by cutting the plants with scissors. We washed the roots twice under tap water to remove soil particles, and then dried the samples with paper towels. This process took roughly 30 s. All shoot and root samples were then immediately stored in paper bags on dry ice to stop further metabolism. This resulted in a total of 20 shoot and 20 root samples per species and experiment.

After one additional week of herbivory (see above), we harvested the remaining five microcosms per diversity level in the PPI experiment and five microcosms per soil legacy level‐planted species combination in the PSI experiment (Tables A1 and A2). We sampled the foliar tissue of one randomly selected control and one randomly selected induced plant individual per species and microcosm by cutting the plants ca. 1 cm above the ground. All samples were then immediately stored in paper bags on dry ice. This resulted in a total of 20 control and 20 induced samples per species and experiment.

In the lab, all samples were stored in a −80°C freezer, and subsequently, freeze‐dried (LABCONCO FreeZone Plus 12 Liter) for 72 h. Dried samples were stored in ziplock bags filled with silica gel at room temperature until we had ground each sample to a fine homogenous powder using a ball mill (Retsch mixer mill MM 400).

### 2.3 Metabolome extraction and analysis

We extracted and analyzed all samples according to Ristok et al. (2019) with slight changes. We extracted 20 mg dried and ground plant tissue of each sample in 1 mL of extraction buffer (methanol/50 mM acetate buffer, pH 4.8; 50/50 [v/v]). All samples were homogenized for 5 min at 30 Hz using a Retsch mixer mill MM 400, and subsequently centrifuged for 10 min at 20,000 *g* and 4°C. We collected the supernatant in a 2 mL Eppendorf tube, repeated the extraction procedure with the remaining pellet, and combined both supernatants. Lastly, we centrifuged (20,000 *g*, 5 min, 4°C) all extracts, transferred 200 μL to an HPLC vial, and added 800 μL extraction buffer, resulting in a 1:5 dilution.

We performed chromatographic separation of all diluted extracts by injecting 2 μL on a Thermo Scientific Dionex UltiMate 3000 (Thermo Scientific Dionex) UPLC unit, equipped with a C18 column (Acclaim RSLC 120 C18, 2.2 μm, 120 Å, 2.1 × 150 mm, Thermo Fisher Scientific). We applied the following binary elution gradient at a flow rate of 0.4 mL min^−1^ and a column temperature of 40°C: 0–2 min, 95% A (water and 0.05% formic acid), 5% B (acetonitrile and 0.05% formic acid); 2–12 min, 5 to 50% B; 12–13 min, 50 to 95% B; 13–15 min, 95% B; 15–16 min, 95 to 5% B; 16–20 min, 5% B.

Metabolites were analyzed on a liquid chromatography quadrupole time‐of‐flight mass spectrometer (LC‐qToF‐MS; Bruker maXis impact HD; Bruker Daltonik) with an electrospray ionization source operated in negative mode. Instrument settings were as follows: capillary voltage, 2500 V; nebulizer, 2.5 bar; dry gas temperature, 220°C; dry gas flow, 11 L min^−1^; scan range, 50–1400 m/z; acquisition rate, 3 Hz. We used sodium formate clusters (10 mM solution of NaOH in 50 / 50% [v/v] isopropanol/water containing 0.2% formic acid) to perform mass calibration.

### 2.4 LC–MS data processing

We followed the LC–MS data processing protocol described in Ristok et al. (2019) with minor changes. We converted the LC‐qToF‐MS raw data to the mzXML format by using the CompassXport utility of the DataAnalysis vendor software. We then trimmed each data file by excluding the same non‐informative regions at the beginning and end of each run using the msconvert function of ProteoWizard v3.0.10095 (Chambers et al., 2012). We performed peak picking, feature alignment, and feature group collapse in R v3.3.3 (R Core Team, 2020) using the Bioconductor (Huber et al., 2015) packages “xcms” (Benton et al., 2010; Smith et al., 2006; Tautenhahn et al., 2008) and “CAMERA” (Kuhl et al., 2012). We used the following “xcms” parameters: peak picking method “centWave” (snthr = 10; ppm = 5; peakwidth = 4, 10); peak grouping method “density” (minfrac = 0.75; bw = 6, 3; mzwid = 0.01); retention time correction method “symmetric”. We used “CAMERA” to annotate adducts, fragments, and isotope peaks with the following parameters: extended rule set (https://gitlab.com/R_packages/chemhelper/‐/tree/master/inst/extdata); perfwhm = 0.6; calcIso = TRUE; calcCaS = TRUE, graphMethod = lpc. Finally, we collapsed each annotated feature group, hereafter referred to as “metabolite” which is described by mass‐to‐charge ratio (*m*/*z*) and retention time (rt), using a maximum heuristic approach (Ristok et al., 2019). The intensity of each metabolite was subsequently normalized to the amount of dried ground plant tissue extracted. We processed all data separately for each experiment, species, and tissue.

### 2.5 Statistical analysis

We analyzed and plotted our data in the statistical software R v4.0.3 (R Core Team, 2020) (http://www.r‐project.org) using the packages “DESeq2” (Love et al., 2014), “vegan” (Oksanen et al., 2020), “mixOmics” (Rohart et al., 2017), and “ggplot2” (Wickham, 2016). Given the nature of our experimental setup, we performed all statistical analyses separately for each experiment, species, and tissue.

We tested for the overall differences in constitutive foliar and root metabolome as well as herbivore‐induced metabolome composition among the plant diversity or soil legacy levels by calculating permutational multivariate analyses of variance using distance matrices. We log +1 transformed the metabolite intensity data to achieve multivariate normality, and used Bray–Curtis dissimilarity to calculate the distance matrices. All analyses were permuted 9999 times. We visualized the results using Partial Least Squares – Discriminant Analysis plots. We used the same approach to test for the differences in the foliar metabolome composition between control and induced plants. We calculated each analysis separately for each species and experiment.

To test for the regulation of metabolites, we calculated differential expression analyses between the monoculture treatment level and each plant diversity or soil legacy mixture level. We used the “DESeq” function provided by the “DESeq2” package with default argument structure and values. Prior to our calculation, missing values were set to zero. We defined a metabolite to be significantly upregulated when the log2 fold change was above 0.6 and the p‐value below 0.05 in comparison to the control. Conversely, we defined a metabolite to be significantly downregulated when the log2 fold change was below −0.6 and the p‐value below 0.05 in comparison to the control. We used the same approach to test for the regulation of metabolites between control and induced plants. We calculated each analysis separately for each species and experiment.

Subsequently, we assigned the putative molecular formula (https://www.chemcalc.org/mf‐finder) and compound name (https://pubchem.ncbi.nlm.nih.gov) based on the high‐resolution mass‐to‐charge values generated by liquid chromatography quadrupole time‐of‐flight mass spectrometry for 95 out of 362 up‐or down regulated metabolites. In cases where our search query returned multiple candidate compounds, we limited the selection to compounds with a mass difference of less than 2 ppm and a verified description in at least one plant species.

## 3 RESULTS

### 3.1 Plant diversity and soil legacy effects on plant metabolomes

**FIGURE 1:**
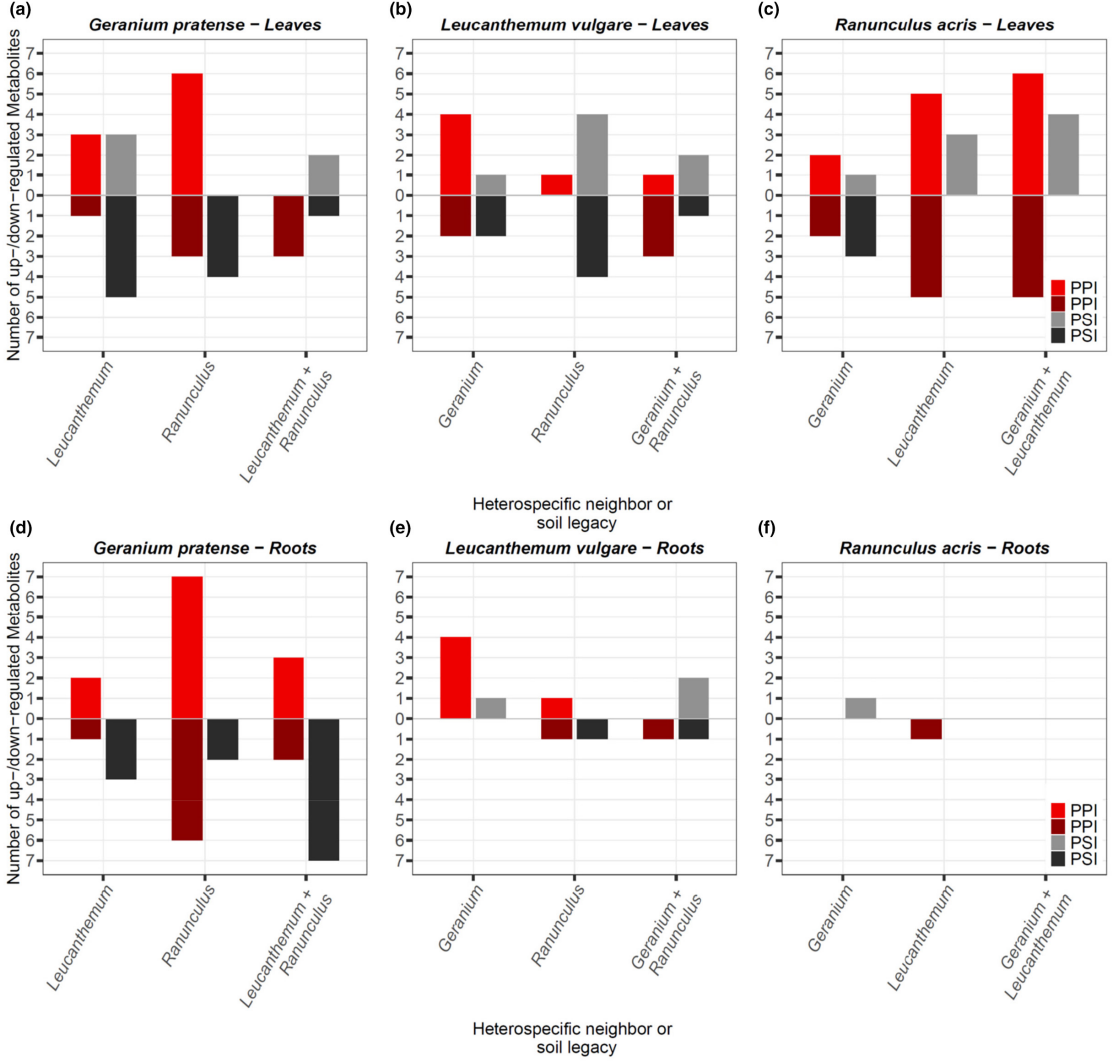
The total number of up‐ and downregulated metabolites in leaves and roots of (a, d) *Geranium pratense*, (b, e) *Leucanthemum vulgare*, and (c, f) *Ranunculus acris* plants grown in microcosms with different neighbors (PPI) or different soil legacies (PSI). The number depicted is in comparison to the monoculture diversity/soil legacy level. Data collected as part of the plant–plant interaction (PPI) experiment are displayed in light red (up) and dark red (down). Data collected as part of the plant–soil interaction (PSI) experiment are displayed in gray (up) and black (down). *Geranium*, *Geranium pratense*; *Leucanthemum*, *Leucanthemum vulgare*; *Ranunculus*, *Ranunculus acris*.

Neither plant diversity nor soil legacy had a significant effect on overall foliar or root metabolome composition (Table 1). However, when we compared metabolomes of plants grown in monocultures with conspecifics growing in mixtures, we discovered a total of 139 significantly up‐ or downregulated metabolites in both leaves and roots (Figure 1). Across both experiments, we found that more foliar than root metabolites were regulated in response to heterospecific plant–plant and plant–soil interactions in *Leucanthemum vulgare* (25 vs. 12) and *Ranunculus acris* (36 vs. 2; Figure 1). Only in *Geranium pratense* were the metabolites in leaves (31 regulated metabolites) and roots (33 regulated metabolites) similarly responsive to heterospecific plant–plant or plant–soil interactions. Overall, metabolites in the leaves of *R. acris* were most responsive, followed by roots and leaves of *G. pratense*, and leaves of *L. vulgare*. Plant–plant interactions generally up‐ and downregulated metabolites across all species, while plant–soil interactions mostly downregulated metabolites in leaves and roots of *G. pratense*, but upregulated metabolites in leaves of *R. acris* (Figure 1).

**TABLE 1:** Differences in the species‐specific foliar, root, and induced metabolome composition among the diversity/soil legacy levels.

Species	Plant–plant interaction experiment									Plant–soil interaction experiment								
	Foliar metabolome composition			Root metabolome composition			Induced metabolome composition			Foliar metabolome composition			Root metabolome composition			Induced metabolome composition		
	*F*	*p*	*R* ^2^	*F*	*p*	*R* ^2^	*F*	*p*	*R* ^2^	*F*	*p*	*R* ^2^	*F*	*p*	*R* ^2^	*F*	*p*	*R* ^2^
*Geranium pratense*	0.858	.803	.092	0.920	.603	.098	0.625	.991	.068	1.176	.155	.122	1.265	.167	.130	1.121	.234	.117
*Leucanthemum vulgare*	1.051	.359	.110	0.706	.933	.077	0.922	.700	.098	1.007	.434	.106	0.770	.895	.088	1.121	.220	.116
*Ranunculus acris*	0.995	.486	.105	0.901	.634	.096	1.167	.216	.121	0.916	.662	.097	0.710	.766	.077	0.903	.703	.096

*Note*: Statistical parameters resulting from a permutational multivariate analysis of variance using distance matrices. We used Bray–Curtis dissimilarity matrices and 9999 permutations.

Abbreviations: *F*, pseudo‐*F*‐value; *p*, *p*‐value.

**FIGURE 2:**
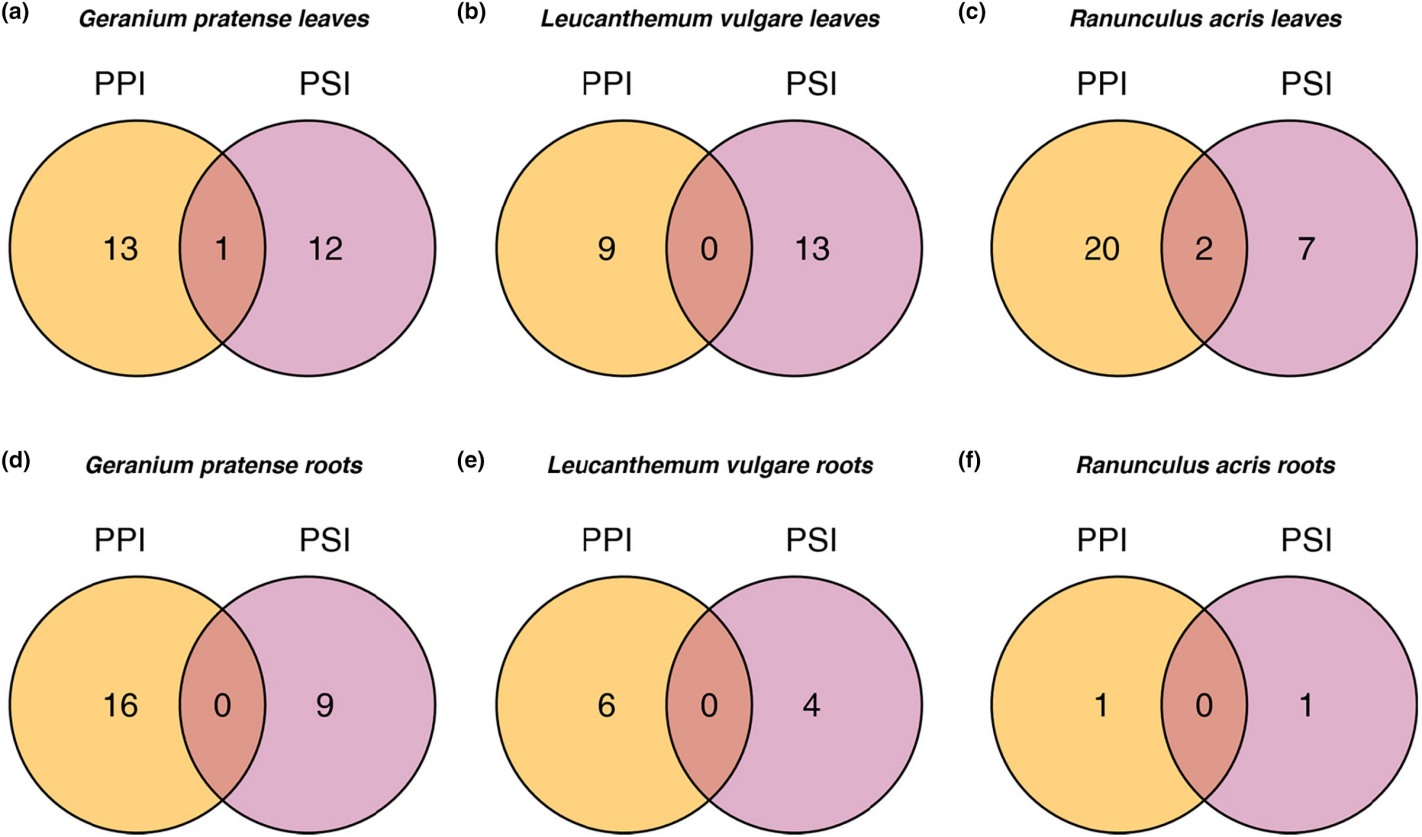
The total number of metabolites in (a–c) leaves or (d–f) roots that were uniquely up‐ and downregulated in plants grown in microcosms with different neighbors (PPI) or different soil legacies (PSI). Metabolites uniquely regulated in the plant–plant interaction (PPI) experiment are depicted in orange. Metabolites uniquely regulated in plant–soil interaction (PSI) experiment are depicted in violet. Overlapping areas indicate the number of up‐ and downregulated metabolites in both experiments. The number depicted is in comparison to the monoculture diversity/soil legacy level.

We found that most regulated metabolites were uniquely synthesized by a plant in response to either plant–plant or plant–soil interactions (Figure 2). This pattern was true across leaves and roots, and across plant species. The only exceptions to this pattern occurred in leaves of *G. pratense* and *R. acris*. Here, we detected metabolites that were regulated in response to both plant–plant and plant–soil interactions (Figure 2). Given that Figure 2 compares the unique and shared regulated metabolites between PPI and PSI across all plant–plant interactions and plant–soil interactions, it may occur that any given enumeration in Figure 2 is lower than in Figure 1, which represents the number of regulated metabolites when a given plant is grown in a specific plant–plant interaction and specific plant–soil interaction that is different from its conspecific control.

**FIGURE A1:**
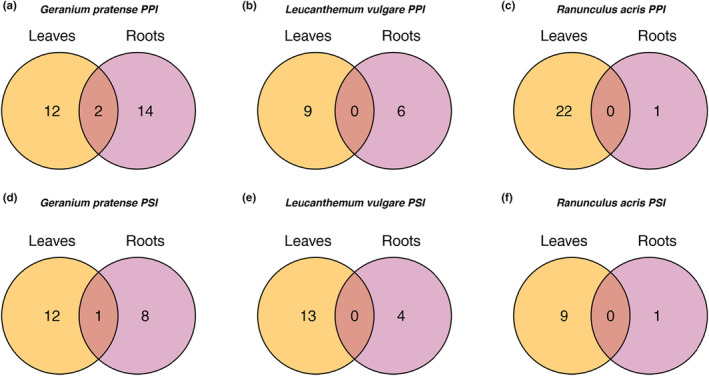
The total number of up‐ and downregulated metabolites in plants grown in microcosms with (a–c) different neighbors (PPI) or (d–f) different soil legacies (PSI). Metabolites uniquely regulated in leaves are depicted in orange. Metabolites uniquely regulated in roots are depicted in violet. Overlapping areas indicate the number of up‐ and downregulated metabolites in both tissues. The number depicted is in comparison to the monoculture diversity/soil legacy level. PPI, plant–plant interaction experiment; PSI, plant–soil interaction experiment.

Moreover, we observed that plants grown either in plant–plant or plant–soil interaction, synthesized and regulated unique metabolites in leaves and roots (Figure A1). The regulated metabolites that we could tentatively assign a molecular formula and compound class or name to, mostly belonged to phenolics, in particular flavonoids, their precursors, and derivatives (Table 2).

**TABLE 2:** Up‐ and downregulated metabolites tentatively assigned in leaves and roots of *Geranium pratense*, *Leucanthemum vulgare*, and *Ranunculus acris*.

Source	Rt [s]	Mass‐to‐charge [*m*/*z*]	Putative compound	Molecular formula	eV	MS fragment	Regulated by		Tissue		Modulated by herbivory
							PPI	PSI	Leaves	Roots	
*Geranium pratense*	64	173.045	Shikimic acid	C_7_H_9_O_5_	35	93, 111, 137, 155		×	×		
	120	169.014	Gallic acid	C_7_H_5_O_5_	35	123, 141	×			×	
	124	483.078	Di‐Gallic acid glycoside	C_20_H_19_O_14_	35		×			×	
	191	483.078	Di‐Gallic acid glycoside	C_20_H_19_O_14_	35		×			×	
	220	305.066	Flavonoid	C_15_H_13_O_7_	35		×		×		
	291	635.089	Flavonoid diglycoside	C_27_H_23_O_18_	35		×	×	×		
	297	609.145	Flavonoid diglycoside	C_27_H_29_O_16_	35			×	×		
	298	577.135	Flavonoid diglycoside	C_30_H_25_O_12_	35		×			×	
	308	299.077	Salicylate glycoside	C_13_H_15_O_8_	35			×	×		
	310	483.078	Di‐Gallic acid glycoside	C_20_H_19_O_14_	35		×			×	
	315	577.134	Flavonoid diglycoside	C_30_H_25_O_12_	35	343		×		×	
	324	627.156	Flavonoid diglycoside	C_27_H_31_O_17_	35	440		×	×		×
	328	289.072	Phenolic acid derivative	C_15_H_13_O_6_	35	179, 245	×	×	×	×	
	389	625.141	Flavonoid diglycoside	C_27_H_29_O_17_	35		×		×		
	393	477.104	Flavonoid glycoside	C_22_H_21_O_12_	35	387	×		×		×
	400	507.114	Flavonoid glycoside	C_23_H_23_O_13_	35		×		×		×
	404	479.083	Flavonoid glycoside	C_21_H_19_O_13_	35			×	×		
	409	667.151	Flavonoid diglycoside	C_29_H_31_O_18_	35		×		×		
	475	447.093	Flavonoid glycoside	C_21_H_19_O_11_	35	183, 335	×		×		
	492	417.082	Flavonoid glycoside	C_20_H_17_O_10_	35			×	×		×
	520	431.097	Flavonoid glycoside	C_21_H_19_O_10_	35		×	×	×		
	532	459.092	Flavone glycoside	C_22_H_19_O_11_	35			×	×		
*Leucanthemum vulgare*	185	315.072	Dihydroxybenzoic acid glucoside	C_13_H_15_O_9_	35			×	×		
	270	353.087	Caffeoylquinic acid	C_16_H_17_O_9_	35	177, 191	×		×		
	277	375.129	Iridoid	C_16_H_23_O_10_	35		×	×	×		×
	281	163.040	Phenolic acid derivative	C_9_H_7_O_3_	35		×		×		
	284	315.071	Dihydroxybenzoic acid glucoside	C_13_H_15_O_9_	35			×	×		
	307	137.024	Salicylate	C_7_H_5_O_3_	35			×	×		
	321	341.088	Caffeic acid glycoside	C_15_H_17_O_9_	35			×		×	
	333	163.040	Phenolic acid derivative	C_9_H_7_O_3_	35		×		×		
	336	353.087	Caffeoylquinic acid	C_16_H_17_O_9_	35	173, 191, 319, 351	×	×	×		
*Leucanthemum vulgare*
	383	551.104	Flavonoid glycoside	C_24_H_23_O_15_	35	507	×		×		×
	413	325.092	Phenolic acid glycoside	C_15_H_17_O_8_	35		×		×		
	420	535.109	Flavonoid glycoside	C_24_H_23_O_14_	35	491	×		×		
	461	223.061	Phenolic acid derivative	C_11_H_11_O_5_	35			×	×		×
	462	336.108	Alkaloid glycoside	C_16_H_18_NO_7_	35	230		×	×		×
	463	505.098	Anthocyanin glycoside	C_23_H_21_O_13_	35			×	×		
	465	591.172	Flavonoid diglycoside	C_28_H_31_O_14_	35		×			×	
	477	625.141	Flavonoid diglycoside	C_27_H_29_O_17_	35	359, 415, 581	×		×		
	484	515.119	Dicaffeoylquinate	C_25_H_23_O_12_	35	353		×	×		
	510	461.109	Flavonoid glycoside	C_22_H_21_O_11_	35		×		×		×
	536	193.050	Phenolic acid derivative	C_10_H_9_O_4_	35			×	×	×	
	540	693.167	Flavonoid diglycoside	C_31_H_33_O_18_	35		×		×		×
	573	163.076	Phenolic acid derivative	C_10_H_11_O_2_	35		×		×		
577	655.188	Flavonoid diglycoside	C_29_H_35_O_17_	35		×		×		×
*Ranunculus acris*	197	197.045	Phenolic acid derivative	C_9_H_9_O_5_	35	135, 151, 179	×		×		×
	260	181.050	Phenolic acid derivative	C_9_H_9_O_4_	35	122	×		×		×
	289	341.088	Caffeic acid glycoside	C_15_H_17_O_9_	35	161, 179, 203	×		×		
	311	137.024	Salicylate	C_7_H_5_O_3_	35		×	×	×		×
	319	465.103	Flavonoid glycoside	C_21_H_21_O_12_	35	277	×		×		
	325	725.193	Flavonoid diglycoside	C_32_H_37_O_19_	35		×		×		
	325	353.087	Caffeoylquinic acid	C_16_H_17_O_9_	35		×		×		
	333	163.040	Phenolic acid derivative	C_9_H_7_O_3_	35		×		×		
	335	325.093	Phenolic acid glycoside	C_15_H_17_O_8_	35	145	×	×	×		
	341	623.160	Flavonoid diglycoside	C_28_H_31_O_16_	35			×	×		×
	351	325.093	Phenolic acid glycoside	C_15_H_17_O_8_	35	145	×	×	×		×
	355	179.035	Acetylsalicylate	C_9_H_7_O_4_	35	135	×	×	×		
	361	695.183	Flavonoid diglycoside	C_31_H_35_O_18_	35		×		×		
	385	317.066	Flavonoid	C_16_H_13_O_7_	35	255	×		×		×
	392	325.092	Phenolic acid glycoside	C_15_H_17_O_8_	35			×	×		×
	398	447.093	Flavonoid glycoside	C_21_H_19_O_11_	35		×	×	×		
	407	699.178	Flavonoid diglycoside	C_30_H_35_O_19_	35		×		×		
	419	577.155	Flavonoid diglycoside	C_27_H_29_O_14_	35			×	×		×
*Ranunculus acris*
	435	669.166	Flavonoid diglycoside	C_29_H_33_O_18_	35		×	×	×		
	438	579.135	Flavonoid diglycoside	C_26_H_27_O_15_	35		×		×		×
	443	595.166	Flavonoid diglycoside	C_27_H_31_O_15_	35	529		×	×		×
	446	667.151	Flavonoid diglycoside	C_29_H_31_O_18_	35	593		×	×		×
	446	449.108	Flavonoid glycoside	C_21_H_21_O_11_	35	287		×	×		×
	448	193.050	Phenolic acid derivative	C_10_H_9_O_4_	35			×	×		×
	449	447.092	Flavonoid glycoside	C_21_H_19_O_11_	35			×	×		×
	449	289.072	Phenolic acid derivative	C_15_H_13_O_6_	35		×		×		
	457	303.051	Flavonoid	C_15_H_11_O_7_	35	125, 177, 259, 275, 285	×		×		
	465	451.124	Flavonoid glycoside	C_21_H_23_O_11_	35	355	×		×		×
	477	577.156	Flavonoid diglycoside	C_27_H_29_O_14_	35		×		×		
	497	331.082	Flavonoid	C_17_H_15_O_7_	35		×		×		×
	507	165.055	Phenolic acid derivative	C_9_H_9_O_3_	35	147	×		×		
581	285.040	Flavonoid	C_15_H_9_O_6_	35		×		×		×

*Note*: We assigned the molecular formula and the putative compound name based on the high‐resolution mass‐to‐charge values generated by liquid chromatography quadrupole time‐of‐flight mass spectrometry.

Abbreviations: eV, fragmentation energy in electron volt; MS, mass spectrometry; PPI, plant–plant interaction; PSI, plant–soil interaction; Rt, retention time in liquid chromatography in seconds.

### 3.2 Plant diversity and soil legacy effects on herbivore‐induced responses.

**FIGURE 3:**
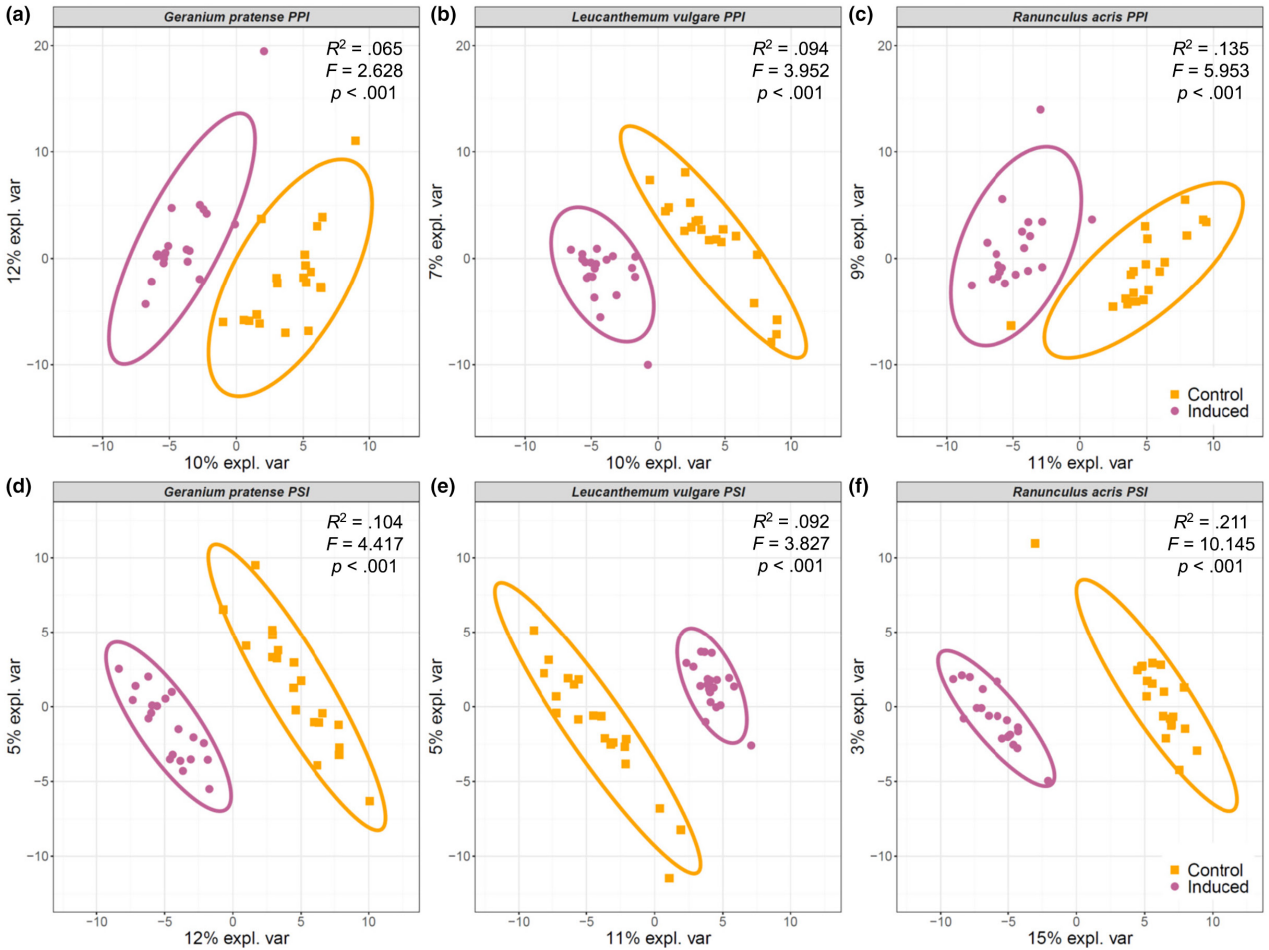
Per species Partial Least Squares – Discriminant Analysis plots of the metabolites found in the foliar metabolomes of *Geranium pratense*, *Leucanthemum vulgare*, and *Ranunculus acris* control or herbivore‐induced plants as part of the (a–c) plant–plant interaction experiment and (d–f) plant–soil interaction experiment. Control plants are displayed in orange squares. Induced plants are displayed in violet circles. Ellipses represent the 95% confidence interval. The metabolite intensity matrix was log + 1 transformed for the purpose of data normalization. Statistical parameters resulting from a permutational multivariate analysis of variance using distance matrices. expl. var, explained variance; *F*, pseudo‐*F*‐value; *p*, *p*‐value.

**FIGURE A2:**
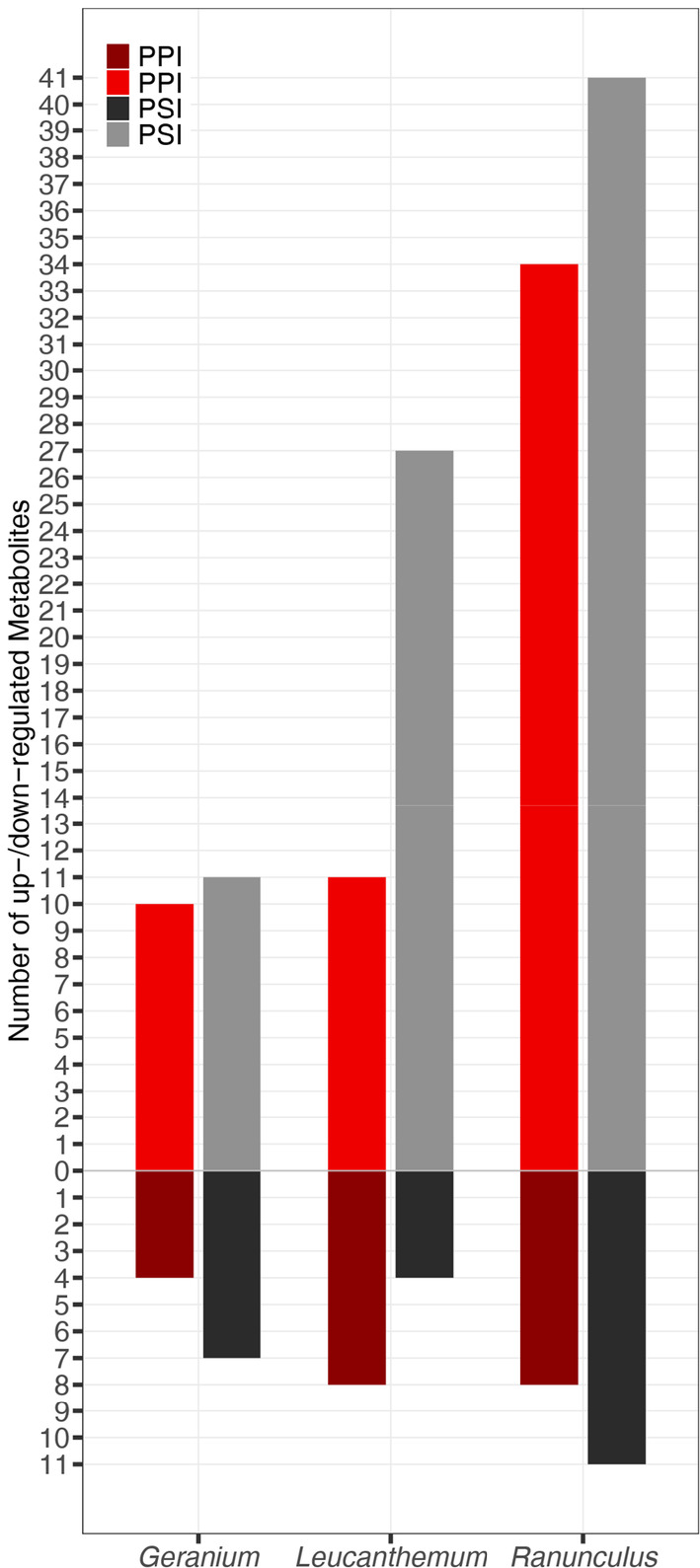
The total number of up‐ and downregulated metabolites in leaves of herbivore‐induced plants. The plants were subjected to 7 days of frass by three 2nd instar *Spodoptera exigua* larvae each. The number depicted is in comparison to control plants grown in similar soil or plant diversity levels, but without herbivore damage. Data collected as part of the plant–plant interaction (PPI) experiment are displayed in light red (up) and dark red (down). Data collected as part of the plant–soil interaction (PSI) experiment are displayed in gray (up) and black (down). *Geranium*, *Geranium pratense*; *Leucanthemum*, *Leucanthemum vulgare*; *Ranunculus*, *Ranunculus acris*.

Both in the PPI (Figure 3a–c) and the PSI (Figure 3d–f) experiment, we discovered significant differences in the foliar metabolome composition across all plant diversity levels and soil legacies between control and herbivore‐induced plants in all plant species. When we tested for the regulation of metabolites between control and induced plants, we found that the total number of upregulated metabolites was higher than the total number of downregulated metabolites across all species (Figure A2). Furthermore, we observed that the absolute number of regulated metabolites was highest when plants had grown in different soil legacies in the PSI experiment. This effect was strongest for *L. vulgare*, while *R. acris* showed the overall strongest response in numbers of regulated metabolites in both the PPI and PSI experiment (Figure A2).

**FIGURE 4:**
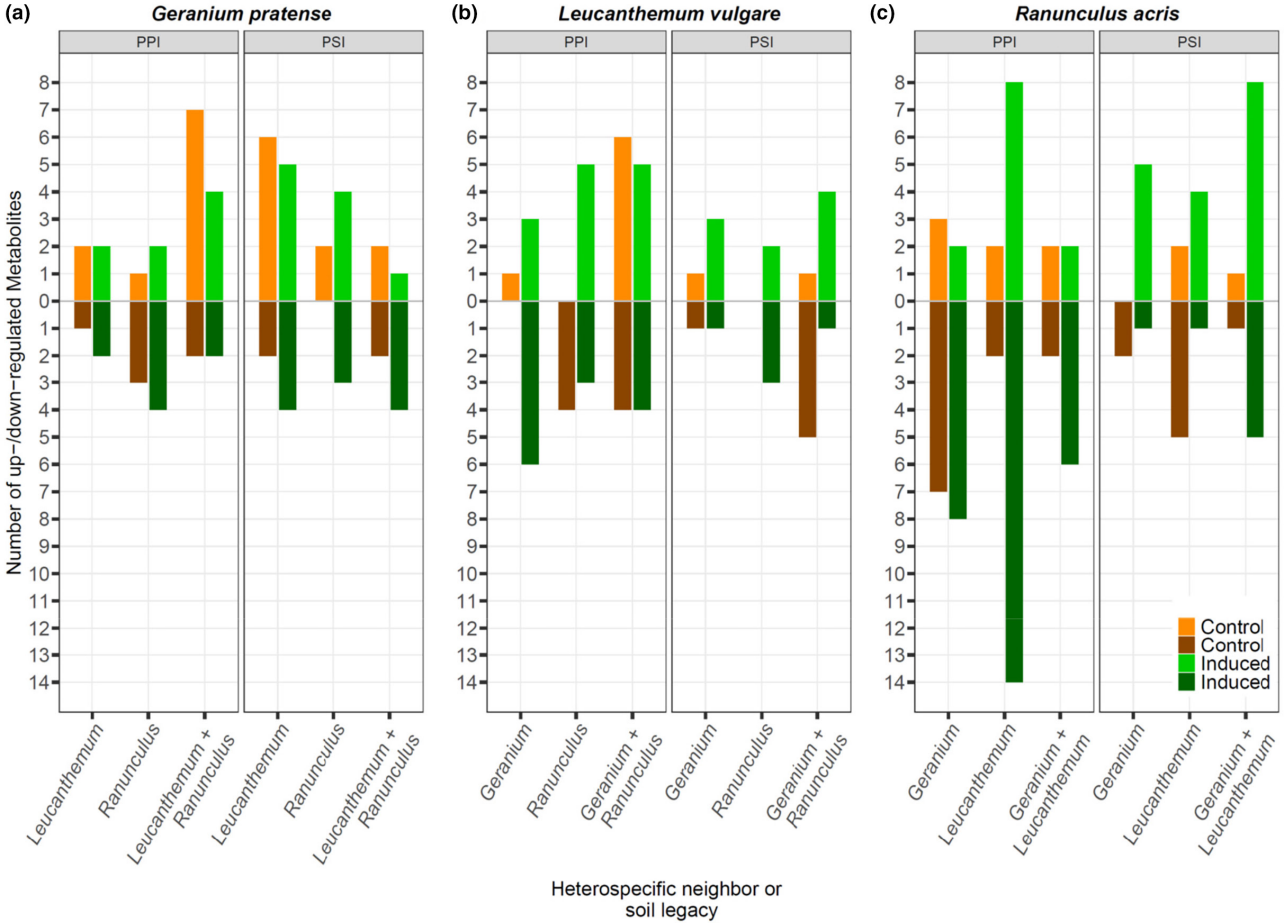
The total number of up‐ and downregulated metabolites in leaves of (a) *Geranium pratense*, (b) *Leucanthemum vulgare*, and (c) *Ranunculus acris* control and herbivore‐induced plants grown in microcosms with different neighbors (PPI) or different soil legacies (PSI). The number depicted is in comparison to the monoculture diversity/soil legacy level. Data collected in control plants are displayed in light orange (up) and dark orange (down). Data collected in induced plants are displayed in light green (up) and dark green (down). Induced plants were infested with *Spodoptera exigua* larvae for 7 days prior to sampling. *Geranium*, *Geranium pratense*; *Leucanthemum*, *Leucanthemum vulgare*; PPI, plant–plant interaction experiment; PSI, plant–soil interaction experiment; *Ranunculus*, *Ranunculus acris*.

**FIGURE A3:**
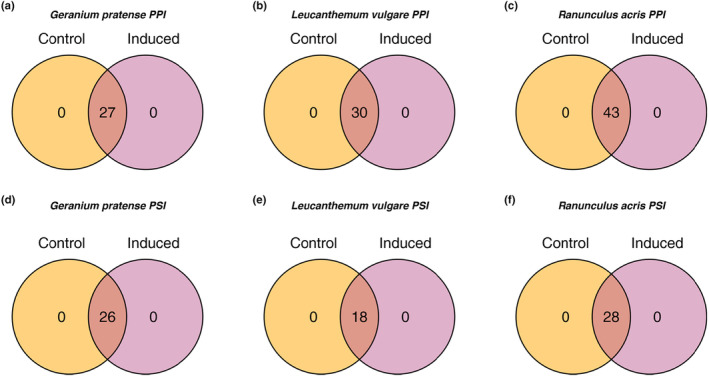
The total number of up‐ and downregulated metabolites in leaves of control and herbivore‐induced plants grown in microcosms with (a–c) different neighbors (PPI) or (d–f) different soil legacies (PSI). Metabolites uniquely regulated in control plants are depicted in orange. Metabolites uniquely regulated in herbivore‐induced plants are depicted in violet. Overlapping areas indicate the number of up‐ and downregulated metabolites in both control and herbivore‐induced plants. The number depicted is in comparison to the monoculture diversity/soil legacy level. PPI, plant–plant interaction experiment; PSI, plant–soil interaction experiment.

In contrast, we found no significant effect of plant diversity in the PPI experiment and of soil legacy in the PSI experiment on the induced metabolome in either species (Table 1). However, when we compared foliar metabolomes of herbivore‐induced plants grown in monocultures with conspecifics growing in mixtures, we discovered a total of 141 significantly up‐ or downregulated metabolites (Figure 4). Both heterospecific plant–plant and plant–soil interactions affected the induction of metabolites compared to conspecific plant–plant or plant–soil interactions. Overall, heterospecific plant–plant interactions regulated more induced metabolites than plant–soil interactions in leaves of *L. vulgare* (26 vs. 14) and *R. acris* (40 vs. 24). In comparison, heterospecific plant–soil interactions had a stronger effect on the regulation of herbivore‐induced metabolites in leaves of *G. pratense* than heterospecific plant–plant interactions (21 vs. 16; Figure 4). In *R. acris*, we discovered that heterospecific plant–plant and plant–soil interactions had contrasting effects on the regulation of induced metabolites. Heterospecific plant–plant interactions strongly downregulated the induction of metabolites, while plant–soil interactions strongly upregulated the induction of metabolites (Figure 4). In contrast, these modulating effects of heterospecific plant–plant or plant–soil interactions on the induction of metabolites were mostly similar or less pronounced in herbivore‐induced plants of *G. pratense* or *L. vulgare* (Figure 4). Across all species and both experiments, we found no de‐novo regulated metabolites in herbivore‐induced plants (Figure A3); all up‐ and downregulated metabolites were present in control plants as well. Similar to the analysis of regulated metabolites in leaves and roots, the tentatively assigned metabolites in herbivore‐induced plants mostly belonged to the family of phenolics, in particular flavonoids, their precursors, and derivatives. Besides, we tentatively assigned two metabolites in *L. vulgare* as an iridoid and an alkaloid glycoside (Table 2).

## 4 DISCUSSION

Our study highlights that both plant–plant interactions and plant–soil interactions can affect foliar and root metabolomic profiles via the regulation of specific metabolites. We showed that metabolites that were regulated in leaves differ from those in roots, and that for two of our three plant species the number of regulated metabolites in leaves was higher than in roots. These results partially confirm our first hypothesis that both plant diversity and soil legacy can alter the overall plant metabolome, as well as affect the regulation of specific metabolites. Moreover, we revealed that the herbivore‐induced metabolomic response is modulated by plant–plant and plant–soil interactions. This strongly suggests that the type and diversity of biotic interactions in the environment can alter induced responses to herbivores in plants. This confirms our second hypothesis that the induced metabolomic response to herbivory is differently affected by plant diversity and soil legacy. Compared to previous studies that focused on plant diversity effects in a field experiment (e.g., Scherling et al., 2010) or plant–soil feedback effects (Huberty et al., 2020; e.g., Ristok et al., 2019), our study provides new insights toward disentangling plant and soil diversity effects on plant metabolomes, and thus plant‐herbivore interactions.

### 4.1 Plant diversity and soil legacy effects on plant metabolomes

While we did not find any overall changes in the foliar or root metabolome composition in response to plant diversity and soil legacy, we observed the unique regulation of 139 metabolites. This is in line with previous work showing that plant diversity or soil legacy can affect the regulation of foliar metabolites (Huberty et al., 2020; Scherling et al., 2010). Our study not only adds to this body of literature but also expands our knowledge by revealing that plant–plant and plant–soil interactions also affect the regulation of root metabolites.

Plant–plant and plant–soil interactions can range from positive, over neutral, to negative (Barry et al., 2019; Cortois et al., 2016). While to our knowledge no study has yet analyzed the effects of positive plant–plant interactions on plant metabolites, in particular negative plant–plant interactions, such as competition, can affect the regulation of metabolites. In our study, we detected 45 metabolites that were significantly upregulated and 36 metabolites that were significantly downregulated as a response to plant–plant interactions. This shift in regulation is potentially a consequence of competition for resources, such as light, nutrients, and water, that can force the plant to either invest resources into growth or defense, as well as affect the production of allelopathic metabolites (Fernandez et al., 2016; Treutter, 2006). Positive plant–soil interactions with mutualists, such as arbuscular mycorrhizal fungi and plant growth‐promoting bacteria, that can improve nutrient uptake and protect against antagonists (Bardgett & van der Putten, 2014; Latz et al., 2012; Wardle et al., 2004), can also affect the regulation of metabolites. In our study, we detected 24 metabolites that were significantly upregulated and 34 metabolites that were significantly downregulated as a response to plant–soil interactions. This shift in regulation may be a response to mycorrhization that, for instance, can affect phenyl alcohol and vitamin associated pathways (Rivero et al., 2015), and/or a response to negative plant–soil interactions with root parasites, pathogens, and herbivores that can reduce root uptake capabilities of resources (Bardgett & van der Putten, 2014; van der Putten et al., 2013). The infection with nematodes, for instance, can affect the regulation of iridoid glycosides (Wurst et al., 2010), while the interaction among different types of soil organisms can further influence the plant metabolome and defense (Lohmann et al., 2009). In addition to these interaction‐specific effects on foliar and root metabolomes, leaves and roots have different functions and are in different abiotic and biotic environments (van Dam, 2009a). These differences are the likely reason that certain metabolite classes in our study, such as alkaloids and phenolics, show different levels of concentration among leaves and roots (Kaplan et al., 2008). Our study confirms that plant–plant and plant–soil interactions affect the regulation of metabolites in leaves and roots. Among the regulated metabolites, we tentatively identified some as flavonoids, iridoids, and alkaloid glycosides. Flavonoids are known as physiologically active compounds, playing important roles as signals in plant–soil biota interactions, as allelochemicals in plant–plant interactions, or as deterrents in plant‐herbivore interactions (Treutter, 2006). Iridoids and alkaloid glycosides are known for their significant roles in plant‐herbivore interactions (Bowers & Puttick, 1988; Mithöfer & Boland, 2008).

We show for the first time that the nature of the regulated metabolites is unique to the tissue and type of biotic interaction, that is, interactions with heterospecific plants and interactions with different soil biota. Hence, our results strongly suggests that plants can adjust their constitutive metabolome, in their roots and their leaves, and specifically react to their biological environment. In light of the recent support of the interaction diversity hypothesis (Whitehead et al., 2021) for the maintenance of chemical diversity, our study presents two potentially additional avenues of biotic interactions (plant–plant and plant–soil interaction) aside from plant‐herbivore interactions that may explain the maintenance of chemical diversity in the plant kingdom. Moreover, our result that the constitutive metabolome in roots and leaves is uniquely shaped by interactions with heterospecific plants and interactions with different soil biota, indicates that prior biotic interaction can affect subsequent biotic interactions, such as with aboveground herbivores.

### 4.2 Plant diversity and soil legacy effects on herbivore‐induced responses

Based on our samples that were taken after 7 days of herbivory to assess the herbivore‐induced metabolome, we also observed alterations in the herbivore‐induced metabolomic response due to plant diversity and soil legacy. Together, plant–plant and plant–soil interactions regulated 82 metabolites in control plants and 141 metabolites in herbivore‐induced plants.

As shown above, plant–plant interactions can modulate growth‐defense trade‐offs that likely vary in strength with changes in plant diversity. In mixed communities, a combination of niche complementarity but increased competition for light, as well as a reduction of herbivory by specialized herbivores via dilution effects, may lead to a higher investment of resources into growth than defense compared to monocultures (Castagneyrol et al., 2014; Eisenhauer et al., 2019; Finch & Collier, 2000; van Moorsel et al., 2018). In fact, earlier work revealed that plants growing in mixed communities invested more resources into growth than defense‐related metabolites compared to plants growing in monoculture (Broz et al., 2010), potentially reducing herbivore resistance. While we did not find differences in the overall metabolome composition of herbivore‐induced plants in response to increasing plant diversity, we observed induced metabolite regulation in mixed communities. Our results suggest that the identity of the neighboring plant species determines the extent and direction of the plant–plant interaction. This has potential consequences for our understanding of plant‐herbivore interactions in mixed communities, but further research is needed to confirm this hypothesis.

Plant–soil interactions, on the other hand, can prepare a plant for future attack, also called priming (Conrath et al., 2006). Systemic priming in plants can occur following interactions with soil microbes, nematodes, and mycorrhizal fungi, allowing the plant to better respond to subsequent herbivory (Kaplan et al., 2008; Martinez‐Medina et al., 2016). While we have not explicitly tested for priming, it may explain why the absolute number of upregulated metabolites in herbivore‐induced plants (in comparison to control plants) was highest when plants had grown in different soil legacies. However, other possible mechanisms, such as systemic acquired resistance to microbial pathogens, exist that could also explain the patterns of metabolite regulation in our study (Ryals et al., 1996).

Finally, we observed differences in the regulation of herbivore‐induced metabolites among our plant species. In *R. acris* plants, plant–plant interactions resulted in a strong downregulation of induced metabolites, while plant–soil interactions resulted in a strong upregulation of induced metabolites. The response to either type of biotic interaction was much more attenuated in *G. pratense* and *L. vulgare*, suggesting differences in the plant species‐specific adaptability which requires future research before general assumptions can be made on the effects of plant diversity versus soil legacy on herbivore resistance.

While the present experiment provides novel insights into how metabolomic profiles, and thereby herbivore resistance, respond to changes in plant and soil biodiversity, it also calls for future studies. To allow for the comparison of plant–plant and plant–soil interactions in our study, we inoculated sterile substrate with liquid field soil inoculum from the Trait‐Based Experiment (Ebeling et al., 2014) in the PSI experiment. This, however, meant that the soil biota communities were adapted and “linked” to the plot‐specific plant communities and that the sand‐peat mixture that was used may have created a different environment than the one the microbes were accustomed to. To fully disentangle plant from soil biodiversity effects on the plant metabolome, one would need to expose plants to artificially constructed soil communities (see e.g., de Souza et al., 2020), also including larger soil organisms (see e.g., Lohmann et al., 2009). In addition, due to space limitations in our greenhouse, we had to run the PPI experiment before the PSI experiment, which slightly affected average temperature and humidity, and we could not set up pots with plant–plant interaction and plant–soil interaction. Future studies may fully randomize their experimental design and add PPI + PSI samples. While this was not feasible in the scope of this study, it would also be important to explore the specific effects of pre‐selected functional soil biota groups, such as nematodes (e.g., Bezemer et al., 2005). Moreover, future studies should explore potential shifts in growth‐defense trade‐offs in more detail by exploring the performance of plants and herbivores. To our knowledge, this kind of comparable experimental design to disentangle plant–plant and plant–soil interaction effects has rarely been employed (but see Kos et al., 2015) and results and conclusions can vary between studies. In addition, future studies may mechanistically test for the effect of PPI and PSI on certain metabolite groups, such as flavonoids, iridoids, and alkaloid glycosides. Hence, we advocate for additional experiments of that kind to generate the necessary data for more reliable conclusions.

## 5 CONCLUSION

Taken together, the present study shows that plant and soil biodiversity trigger unique responses in the plant's metabolomic profile that modulate the induced response to herbivory. By disentangling plant diversity from soil biodiversity effects, we advance our understanding of the mechanisms that shape plant metabolomes and thus, herbivore resistance.

## AUTHOR CONTRIBUTIONS

**Christian Ristok:** Conceptualization (equal); data curation (lead); formal analysis (lead); funding acquisition (equal); investigation (equal); visualization (lead); writing – original draft (lead); writing – review and editing (equal). **Nico Eisenhauer:** Conceptualization (equal); funding acquisition (equal); investigation (equal); resources (equal); supervision (equal); writing – review and editing (equal). **Alexander Weinhold:** Conceptualization (equal); data curation (equal); formal analysis (equal); investigation (equal); supervision (equal); writing – review and editing (equal). **Nicole M. van Dam:** Conceptualization (equal); funding acquisition (equal); investigation (equal); project administration (equal); resources (equal); supervision (equal); writing – review and editing (equal).

## CONFLICT OF INTEREST STATEMENT

The authors declare no competing interest.

## Data Availability

Data available from the Dryad Digital Repository https://doi.org/10.5061/dryad.p2ngf1vx9 (Ristok, Eisenhauer, et al., 2023).

## APPENDIX 1

**TABLE A1:** Overview of the experimental design of the plant–plant‐interaction experiment.

Experimental design plant–plant interaction experiment							
Species pool	*Geranium pratense* (G) – *Leucanthemum vulgare* (L) – *Ranunculus acris* (R)						
Diversity levels	G	L	R	G + L	G + R	L + R	G + L + R
Nr. of plants/microcosm	12	12	12	6 + 6	6 + 6	6 + 6	4 + 4 + 4
Nr. of microcosms	5	5	5	5	5	5	5
Herbivory treatment	×2	×2	×2	×2	×2	×2	×2
Total Nr. of microcosms	10	10	10	10	10	10	10
1st Harvest after 7 weeks of growth							
Nr. of microcosms	5	5	5	5	5	5	5
Nr. of plants sampled/microcosm	1	1	1	1 + 1	1 + 1	1 + 1	1 + 1 + 1
Total Nr. of samples (shoot/root)	5/5	5/5	5/5	G: 5/5 L: 5/5	G: 5/5 R: 5/5	L: 5/5 R: 5/5	G: 5/5 L: 5/5 R: 5/5
2nd Harvest after 1 additional week of herbivory							
Nr. of microcosms	5	5	5	5	5	5	5
Nr. of plants/microcosm (control/induced)	10/2	10/2	10/2	G: 4/2 L: 4/2	G: 4/2 R: 4/2	L: 4/2 R: 4/2	G: 2/2 L: 2/2 R: 2/2
Nr. of plants sampled/microcosm (control/induced)	1/1	1/1	1/1	G: 1/1 L: 1/1	G: 1/1 R: 1/1	L: 1/1 R: 1/1	G: 1/1 L: 1/1 R: 1/1
Total Nr. of samples (control/induced)	5/5	5/5	5/5	G: 5/5 L: 5/5	G: 5/5 R: 5/5	L: 5/5 R: 5/5	G: 5/5 L: 5/5 R: 5/5

**TABLE A2:** Overview of the experimental design of the plant–soil‐interaction experiment.

Experimental design plant–soil interaction experiment							
Species pool	*Geranium pratense* (G) – *Leucanthemum vulgare* (L) – *Ranunculus acris* (R)						
Soil legacy levels	G	L	R	G + L	G + R	L + R	G + L + R
Planted species & nr. of plants/microcosm	G:4	L:4	R:4	G: 4 L: 4	G: 4 R: 4	L: 4 R: 4	G: 4 L: 4 R: 4
Nr. of microcosms	5	5	5	G: 5 L: 5	G: 5 R: 5	L: 5 R: 5	G: 5 L: 5 R: 5
Herbivory treatment	×2	×2	×2	×2	×2	×2	×2
Total Nr. of microcosms	10	10	10	G: 10 L: 10	G: 10 R: 10	L: 10 R: 10	G: 10 L: 10 R: 10
1st Harvest after 7 weeks of growth							
Nr. of microcosms	5	5	5	G: 5 L: 5	G: 5 R: 5	L: 5 R: 5	G: 5 L: 5 R: 5
Nr. of plants sampled/microcosm	1	1	1	G: 1 L: 1	G: 1 R: 1	L: 1 R: 1	G: 1 L: 1 R: 1
Total Nr. of samples (shoot/root)	5/5	5/5	5/5	G: 5/5 L: 5/5	G: 5/5 R: 5/5	L: 5/5 R: 5/5	G: 5/5 L: 5/5 R: 5/5
2nd Harvest after 1 additional week of herbivory							
Nr. of microcosms	5	5	5	G: 5 L: 5	G: 5 R: 5	L: 5 R: 5	G: 5 L: 5 R: 5
Nr. of plants/microcosm (control/induced)	2/2	2/2	2/2	G: 2/2 L: 2/2	G: 2/2 R: 2/2	L: 2/2 R: 2/2	G: 2/2 L: 2/2 R: 2/2
Nr. of plants sampled/microcosm (control/induced)	1/1	1/1	1/1	G: 1/1 L: 1/1	G: 1/1 R: 1/1	L: 1/1 R: 1/1	G: 1/1 L: 1/1 R: 1/1
Total Nr. of samples (control/induced)	5/5	5/5	5/5	G: 5/5 L: 5/5	G: 5/5 R: 5/5	L: 5/5 R: 5/5	G: 5/5 L: 5/5 R: 5/5

## References

[ece310667-bib-0001] Alphei, J. , & Scheu, S. (1993). Effects of biocidal treatments on biological and nutritional properties of a mull‐structured woodland soil. In L. Brussaard & M. J. Kooistra (Eds.), Soil structure/soil biota interrelationships (pp. 435–448). Elsevier.

[ece310667-bib-0002] Baldwin, I. T. , Halitschke, R. , Paschold, A. , von Dahl, C. C. , & Preston, C. A. (2006). Volatile signaling in plant‐plant interactions: “Talking trees” in the genomics era. Science, 311(5762), 812–815. 10.1126/science.1118446 16469918

[ece310667-bib-0003] Bardgett, R. D. , & van der Putten, W. H. (2014). Belowground biodiversity and ecosystem functioning. Nature, 515(7528), 505–511. 10.1038/nature13855 25428498

[ece310667-bib-0004] Bardgett, R. D. , & Wardle, D. A. (2010). Aboveground‐belowground linkages: Biotic interactions, ecosystem processes, and global change. Oxford University Press.

[ece310667-bib-0005] Barry, K. E. , Mommer, L. , van Ruijven, J. , Wirth, C. , Wright, A. J. , Bai, Y. , Connolly, J. , De Deyn, G. B. , de Kroon, H. , Isbell, F. , Milcu, A. , Roscher, C. , Scherer‐Lorenzen, M. , Schmid, B. , & Weigelt, A. (2019). The future of complementarity: Disentangling causes from consequences. Trends in Ecology & Evolution, 34(2), 167–180. 10.1016/j.tree.2018.10.013 30527960

[ece310667-bib-0006] Benton, H. P. , Want, E. J. , & Ebbels, T. M. D. (2010). Correction of mass calibration gaps in liquid chromatography–mass spectrometry metabolomics data. Bioinformatics, 26(19), 2488–2489. 10.1093/bioinformatics/btq441 20671148

[ece310667-bib-0007] Bezemer, T. M. , De Deyn, G. B. , Bossinga, T. M. , Van Dam, N. M. , Harvey, J. A. , & Van der Putten, W. H. (2005). Soil community composition drives aboveground plant–herbivore–parasitoid interactions. Ecology Letters, 8(6), 652–661. 10.1111/j.1461-0248.2005.00762.x

[ece310667-bib-0008] Bezemer, T. M. , Fountain, M. T. , Barea, J. M. , Christensen, S. , Dekker, S. C. , Duyts, H. , van Hal, R. , Harvey, J. A. , Hedlund, K. , Maraun, M. , Mikola, J. , Mladenov, A. G. , Robin, C. , Ruiter, D. , Scheu, S. , Setälä, H. , Šmilauer, P. , & Putten, W. H. (2010). Divergent composition but similar function of soil food webs of individual plants: Plant species and community effects. Ecology, 91(10), 3027–3036. 10.1890/09-2198.1 21058562

[ece310667-bib-0009] Bezemer, T. M. , & van Dam, N. M. (2005). Linking aboveground and belowground interactions via induced plant defenses. Trends in Ecology & Evolution, 20(11), 617–624. 10.1016/j.tree.2005.08.006 16701445

[ece310667-bib-0010] Bowers, M. D. , & Puttick, G. M. (1988). Response of generalist and specialist insects to qualitative allelochemical variation. Journal of Chemical Ecology, 14(1), 319–334. 10.1007/BF01022549 24277012

[ece310667-bib-0011] Broz, A. K. , Broeckling, C. D. , De‐la‐Peña, C. , Lewis, M. R. , Greene, E. , Callaway, R. M. , Sumner, L. W. , & Vivanco, J. M. (2010). Plant neighbor identity influences plant biochemistry and physiology related to defense. BMC Plant Biology, 10(1), 115. 10.1186/1471-2229-10-115 20565801 PMC3095278

[ece310667-bib-0012] Castagneyrol, B. , Jactel, H. , Vacher, C. , Brockerhoff, E. G. , & Koricheva, J. (2014). Effects of plant phylogenetic diversity on herbivory depend on herbivore specialization. Journal of Applied Ecology, 51(1), 134–141. 10.1111/1365-2664.12175

[ece310667-bib-0013] Chambers, M. C. , Maclean, B. , Burke, R. , Amodei, D. , Ruderman, D. L. , Neumann, S. , Gatto, L. , Fischer, B. , Pratt, B. , & Egertson, J. (2012). A cross‐platform toolkit for mass spectrometry and proteomics. Nature Biotechnology, 30(10), 918–920. 10.1038/nbt.2377 PMC3471674 23051804

[ece310667-bib-0014] Conrath, U. , Beckers, G. J. M. , Flors, V. , García‐Agustín, P. , Jakab, G. , Mauch, F. , Newman, M.‐A. , Pieterse, C. M. J. , Poinssot, B. , Pozo, M. J. , Pugin, A. , Schaffrath, U. , Ton, J. , Wendehenne, D. , Zimmerli, L. , & Mauch‐Mani, B. (2006). Priming: Getting ready for battle. Molecular Plant–Microbe Interactions, 19(10), 1062–1071. 10.1094/MPMI-19-1062 17022170

[ece310667-bib-0015] Cortois, R. , Schröder‐Georgi, T. , Weigelt, A. , van der Putten, W. H. , & De Deyn, G. B. (2016). OLD DO NOT USE plant–soil feedbacks: Role of plant functional group and plant traits. Journal of Ecology, 104, 1608–1617. 10.1111/1365-2745.12643

[ece310667-bib-0016] de Souza, R. S. C. , Armanhi, J. S. L. , & Arruda, P. (2020). From microbiome to traits: Designing synthetic microbial communities for improved crop resiliency. Frontiers in Plant Science, 11, 1–7. 10.3389/fpls.2020.01179 32983187 PMC7484511

[ece310667-bib-0017] Ebeling, A. , Pompe, S. , Baade, J. , Eisenhauer, N. , Hillebrand, H. , Proulx, R. , Roscher, C. , Schmid, B. , Wirth, C. , & Weisser, W. W. (2014). A trait‐based experimental approach to understand the mechanisms underlying biodiversity–ecosystem functioning relationships. Basic and Applied Ecology, 15(3), 229–240. 10.1016/j.baae.2014.02.003

[ece310667-bib-0018] Ehrenfeld, J. G. , Ravit, B. , & Elgersma, K. (2005). Feedback in the plant‐soil system. Annual Review of Environment and Resources, 30(1), 75–115. 10.1146/annurev.energy.30.050504.144212

[ece310667-bib-0019] Eisenhauer, N. (2012). Aboveground–belowground interactions as a source of complementarity effects in biodiversity experiments. Plant and Soil, 351(1–2), 1–22. 10.1007/s11104-011-1027-0

[ece310667-bib-0020] Eisenhauer, N. , Bonkowski, M. , Brose, U. , Buscot, F. , Durka, W. , Ebeling, A. , Fischer, M. , Gleixner, G. , Heintz‐Buschart, A. , Hines, J. , Jesch, A. , Lange, M. , Meyer, S. , Roscher, C. , Scheu, S. , Schielzeth, H. , Schloter, M. , Schulz, S. , Unsicker, S. , … Schmid, B. (2019). Biotic interactions, community assembly, and eco‐evolutionary dynamics as drivers of long‐term biodiversity–ecosystem functioning relationships. Research Ideas and Outcomes: The Open Science Journal, 5, e47042. 10.3897/rio.5.e47042

[ece310667-bib-0021] Elvira, S. , Gorría, N. , Muñoz, D. , Williams, T. , & Caballero, P. (2010). A simplified low‐cost diet for rearing *Spodoptera exigua* (Lepidoptera: Noctuidae) and its effect on *S. exigua* nucleopolyhedrovirus production. Journal of Economic Entomology, 103(1), 17–24. 10.1603/EC09246 20214363

[ece310667-bib-0022] Ferlian, O. , Biere, A. , Bonfante, P. , Buscot, F. , Eisenhauer, N. , Fernandez, I. , Hause, B. , Herrmann, S. , Krajinski‐Barth, F. , Meier, I. C. , Pozo, M. J. , Rasmann, S. , Rillig, M. C. , Tarkka, M. T. , van Dam, N. M. , Wagg, C. , & Martinez‐Medina, A. (2018). Growing research networks on mycorrhizae for mutual benefits. Trends in Plant Science, 23(11), 975–984. 10.1016/j.tplants.2018.08.008 30241736 PMC6370000

[ece310667-bib-0023] Fernandez, C. , Monnier, Y. , Santonja, M. , Gallet, C. , Weston, L. A. , Prévosto, B. , Saunier, A. , Baldy, V. , & Bousquet‐Mélou, A. (2016). The impact of competition and allelopathy on the trade‐off between plant defense and growth in two contrasting tree species. Frontiers in Plant Science, 7, 594. 10.3389/fpls.2016.00594 27200062 PMC4855863

[ece310667-bib-0024] Finch, S. , & Collier, R. H. (2000). Host‐plant selection by insects – A theory based on ‘appropriate/inappropriate landings’ by pest insects of cruciferous plants. Entomologia Experimentalis et Applicata, 96(2), 91–102. 10.1046/j.1570-7458.2000.00684.x

[ece310667-bib-0025] Gehring, C. A. , & Whitham, T. G. (1994). Interactions between aboveground herbivores and the mycorrhizal mutualists of plants. Trends in Ecology & Evolution, 9(7), 251–255. 10.1016/0169-5347(94)90290-9 21236843

[ece310667-bib-0026] Hamilton, E. W. , & Frank, D. A. (2001). Can plants stimulate soil microbes and their own nutrient supply? Evidence from a grazing tolerant grass. Ecology, 82(9), 2397–2402. 10.1890/0012-9658(2001)082[2397:CPSSMA]2.0.CO;2

[ece310667-bib-0027] Hol, W. H. G. , de Boer, W. , Termorshuizen, A. J. , Meyer, K. M. , Schneider, J. H. M. , van Dam, N. M. , van Veen, J. A. , & van der Putten, W. H. (2010). Reduction of rare soil microbes modifies plant–herbivore interactions. Ecology Letters, 13(3), 292–301. 10.1111/j.1461-0248.2009.01424.x 20070364

[ece310667-bib-0028] Huber, W. , Carey, V. J. , Gentleman, R. , Anders, S. , Carlson, M. , Carvalho, B. S. , Bravo, H. C. , Davis, S. , Gatto, L. , Girke, T. , Gottardo, R. , Hahne, F. , Hansen, K. D. , Irizarry, R. A. , Lawrence, M. , Love, M. I. , MacDonald, J. , Obenchain, V. , Oleś, A. K. , … Morgan, M. (2015). Orchestrating high‐throughput genomic analysis with Bioconductor. Nature Methods, 12(2), 115–121. 10.1038/nmeth.3252 25633503 PMC4509590

[ece310667-bib-0029] Huberty, M. , Choi, Y. H. , Heinen, R. , & Bezemer, T. M. (2020). Above‐ground plant metabolomic responses to plant–soil feedbacks and herbivory. Journal of Ecology, 108(4), 1703–1712. 10.1111/1365-2745.13394

[ece310667-bib-0030] Kaplan, I. , Halitschke, R. , Kessler, A. , Sardanelli, S. , & Denno, R. F. (2008). Constitutive and induced defenses to herbivory in above‐ and belowground plant tissues. Ecology, 89(2), 392–406. 10.1890/07-0471.1 18409429

[ece310667-bib-0031] Karban, R. , & Baldwin, I. T. (1997). Induced responses to herbivory. University of Chicago Press. https://press.uchicago.edu/ucp/books/book/chicago/I/bo3644508.html

[ece310667-bib-0032] Kardol, P. , Cornips, N. J. , van Kempen, M. M. L. , Bakx‐Schotman, J. M. T. , & van der Putten, W. H. (2007). Microbe‐mediated plant‐soil feedback causes historical contingency effects in plant community assembly. Ecological Monographs, 77(2), 147–162. 10.1890/06-0502

[ece310667-bib-0033] Kos, M. , Bukovinszky, T. , Mulder, P. P. J. , & Bezemer, T. M. (2015). Disentangling above‐ and belowground neighbor effects on the growth, chemistry, and arthropod community on a focal plant. Ecology, 96(1), 164–175. 10.1890/14-0563.1 26236901

[ece310667-bib-0034] Kuhl, C. , Tautenhahn, R. , Böttcher, C. , Larson, T. R. , & Neumann, S. (2012). CAMERA: An integrated strategy for compound spectra extraction and annotation of liquid chromatography/mass spectrometry data sets. Analytical Chemistry, 84(1), 283–289. 10.1021/ac202450g 22111785 PMC3658281

[ece310667-bib-0035] Kulmatiski, A. , Beard, K. H. , Stevens, J. R. , & Cobbold, S. M. (2008). Plant–soil feedbacks: A meta‐analytical review. Ecology Letters, 11(9), 980–992. 10.1111/j.1461-0248.2008.01209.x 18522641

[ece310667-bib-0036] Latz, E. , Eisenhauer, N. , Rall, B. C. , Allan, E. , Roscher, C. , Scheu, S. , & Jousset, A. (2012). Plant diversity improves protection against soil‐borne pathogens by fostering antagonistic bacterial communities. Journal of Ecology, 100(3), 597–604. 10.1111/j.1365-2745.2011.01940.x

[ece310667-bib-0037] Lohmann, M. , Scheu, S. , & Müller, C. (2009). Decomposers and root feeders interactively affect plant defence in *Sinapis alba* . Oecologia, 160(2), 289–298. 10.1007/s00442-009-1306-0 19252930 PMC3085730

[ece310667-bib-0038] Love, M. I. , Huber, W. , & Anders, S. (2014). Moderated estimation of fold change and dispersion for RNA‐seq data with DESeq2. Genome Biology, 15(12), 550. 10.1186/s13059-014-0550-8 25516281 PMC4302049

[ece310667-bib-0039] Martinez‐Medina, A. , Flors, V. , Heil, M. , Mauch‐Mani, B. , Pieterse, C. M. J. , Pozo, M. J. , Ton, J. , van Dam, N. M. , & Conrath, U. (2016). Recognizing plant defense priming. Trends in Plant Science, 21(10), 818–822. 10.1016/j.tplants.2016.07.009 27507609

[ece310667-bib-0040] Mithöfer, A. , & Boland, W. (2008). Recognition of herbivory‐associated molecular patterns. Plant Physiology, 146(3), 825–831. 10.1104/pp.107.113118 18316636 PMC2259064

[ece310667-bib-0041] Oksanen, J. , Blanchet, F. G. , Friendly, M. , Kindt, R. , Legendre, P. , McGlinn, D. , Minchin, P. R. , O'Hara, R. B. , Simpson, G. L. , Solymos, P. , Stevens, M. H. H. , Szoecs, E. , & Wagner, H. (2020). vegan: Community ecology package . https://CRAN.R‐project.org/package=vegan

[ece310667-bib-0042] Oliver, S. G. , Winson, M. K. , Kell, D. B. , & Baganz, F. (1998). Systematic functional analysis of the yeast genome. Trends in Biotechnology, 16(9), 373–378. 10.1016/S0167-7799(98)01214-1 9744112

[ece310667-bib-0043] Peñuelas, J. , & Sardans, J. (2009). Ecological metabolomics. Chemistry and Ecology, 25(4), 305–309. 10.1080/02757540903062517

[ece310667-bib-0044] Peters, K. , Worrich, A. , Weinhold, A. , Alka, O. , Balcke, G. , Birkemeyer, C. , Bruelheide, H. , Calf, O. W. , Dietz, S. , Dührkop, K. , Gaquerel, E. , Heinig, U. , Kücklich, M. , Macel, M. , Müller, C. , Poeschl, Y. , Pohnert, G. , Ristok, C. , Rodríguez, V. M. , … van Dam, N. M. (2018). Current challenges in plant eco‐metabolomics. International Journal of Molecular Sciences, 19(5), 1–38. 10.3390/ijms19051385 PMC5983679 29734799

[ece310667-bib-0045] R Core Team . (2020). R: A language and environment for statistical computing. R Foundation for Statistical Computing. https://www.R‐project.org/

[ece310667-bib-0046] Ristok, C. , Eisenhauer, N. , Weinhold, A. , & van Dam, N. M. (2023). Plant diversity and soil legacy independently affect the plant metabolome and induced responses following herbivory [Dataset]. Dryad. 10.5061/dryad.p2ngf1vx9 PMC10622854 37928199

[ece310667-bib-0047] Ristok, C. , Poeschl, Y. , Dudenhöffer, J.‐H. , Ebeling, A. , Eisenhauer, N. , Vergara, F. , Wagg, C. , van Dam, N. M. , & Weinhold, A. (2019). Plant species richness elicits changes in the metabolome of grassland species via soil biotic legacy. Journal of Ecology, 107(5), 2240–2254. 10.1111/1365-2745.13185

[ece310667-bib-0048] Ristok, C. , Weinhold, A. , Ciobanu, M. , Poeschl, Y. , Roscher, C. , Vergara, F. , Eisenhauer, N. , & van Dam, N. M. (2023). Plant diversity effects on herbivory are related to soil biodiversity and plant chemistry. Journal of Ecology, 111(2), 412–427. 10.1111/1365-2745.14032

[ece310667-bib-0049] Rivero, J. , Gamir, J. , Aroca, R. , Pozo, M. J. , & Flors, V. (2015). Metabolic transition in mycorrhizal tomato roots. Frontiers in Microbiology, 6, 598. 10.3389/fmicb.2015.00598 26157423 PMC4477175

[ece310667-bib-0050] Rohart, F. , Gautier, S. D. , Singh, A. , & Le Cao, K.‐A. (2017). mixOmics: An R package for 'omics feature selection and multiple data integration. PLoS Computational Biology, 13(11), e1005752. 29099853 10.1371/journal.pcbi.1005752 PMC5687754

[ece310667-bib-0051] Ryals, J. , Neuenschwander, U. , Willits, M. , Molina, A. , Steiner, H. , & Hunt, M. (1996). Systemic acquired resistance. The Plant Cell, 8(10), 1809–1819. 12239363 10.1105/tpc.8.10.1809 PMC161316

[ece310667-bib-0052] Scherling, C. , Roscher, C. , Giavalisco, P. , Schulze, E.‐D. , & Weckwerth, W. (2010). Metabolomics unravel contrasting effects of biodiversity on the performance of individual plant species. PLoS One, 5(9), 1–13. 10.1371/journal.pone.0012569 PMC2935349 20830202

[ece310667-bib-0053] Smith, C. A. , Want, E. J. , O'Maille, G. , Abagyan, R. , & Siuzdak, G. (2006). XCMS: Processing mass spectrometry data for metabolite profiling using nonlinear peak alignment, matching, and identification. Analytical Chemistry, 78(3), 779–787. 10.1021/ac051437y 16448051

[ece310667-bib-0054] Steinauer, K. , Chatzinotas, A. , & Eisenhauer, N. (2016). Root exudate cocktails: The link between plant diversity and soil microorganisms? Ecology and Evolution, 6(20), 7387–7396. 10.1002/ece3.2454 28725406 PMC5513276

[ece310667-bib-0055] Tautenhahn, R. , Böttcher, C. , & Neumann, S. (2008). Highly sensitive feature detection for high resolution LC/MS. BMC Bioinformatics, 9, 504. 10.1186/1471-2105-9-504 19040729 PMC2639432

[ece310667-bib-0056] Treutter, D. (2006). Significance of flavonoids in plant resistance: A review. Environmental Chemistry Letters, 4(3), 147–157. 10.1007/s10311-006-0068-8

[ece310667-bib-0057] Trevors, J. T. (1996). Sterilization and inhibition of microbial activity in soil. Journal of Microbiological Methods, 26(1), 53–59. 10.1016/0167-7012(96)00843-3

[ece310667-bib-0058] van Dam, N. M. (2009a). Belowground herbivory and plant defenses. Annual Review of Ecology, Evolution, and Systematics, 40(1), 373–391. 10.1146/annurev.ecolsys.110308.120314

[ece310667-bib-0059] van Dam, N. M. (2009b). How plants cope with biotic interactions. Plant Biology, 11(1), 1–5. 10.1111/j.1438-8677.2008.00179.x 19121108

[ece310667-bib-0060] van Dam, N. M. , & Baldwin, I. T. (2001). Competition mediates costs of jasmonate‐induced defences, nitrogen acquisition and transgenerational plasticity in *Nicotiana attenuata* . Functional Ecology, 15(3), 406–415. 10.1046/j.1365-2435.2001.00533.x

[ece310667-bib-0061] van Dam, N. M. , & Heil, M. (2011). Multitrophic interactions below and above ground: En route to the next level. Journal of Ecology, 99(1), 77–88. 10.1111/j.1365-2745.2010.01761.x

[ece310667-bib-0062] van Dam, N. M. , & van der Meijden, E. (2011). A role for metabolomics in plant ecology. In R. D. Hall (Ed.), Annual plant reviews (Vol. 43, pp. 87–107). Wiley‐Blackwell.

[ece310667-bib-0063] van de Voorde, T. F. J. , van der Putten, W. H. , & Bezemer, T. M. (2011). Intra‐ and interspecific plant–soil interactions, soil legacies and priority effects during old‐field succession. Journal of Ecology, 99(4), 945–953. 10.1111/j.1365-2745.2011.01815.x

[ece310667-bib-0064] Van Der Heijden, M. G. A. , Bardgett, R. D. , & Van Straalen, N. M. (2008). The unseen majority: Soil microbes as drivers of plant diversity and productivity in terrestrial ecosystems. Ecology Letters, 11(3), 296–310. 10.1111/j.1461-0248.2007.01139.x 18047587

[ece310667-bib-0065] van der Putten, W. H. , Bardgett, R. D. , Bever, J. D. , Bezemer, T. M. , Casper, B. B. , Fukami, T. , Kardol, P. , Klironomos, J. N. , Kulmatiski, A. , Schweitzer, J. A. , Suding, K. N. , Van de Voorde, T. F. J. , & Wardle, D. A. (2013). Plant‐soil feedbacks: The past, the present and future challenges. Journal of Ecology, 101(2), 265–276. 10.1111/1365-2745.12054

[ece310667-bib-0066] van Moorsel, S. J. , Hahl, T. , Wagg, C. , De Deyn, G. B. , Flynn, D. F. B. , Zuppinger‐Dingley, D. , & Schmid, B. (2018). Community evolution increases plant productivity at low diversity. Ecology Letters, 21(1), 128–137. 10.1111/ele.12879 29148170

[ece310667-bib-0067] Wardle, D. A. , Bardgett, R. D. , Klironomos, J. N. , Setälä, H. , van der Putten, W. H. , & Wall, D. H. (2004). Ecological linkages between aboveground and belowground biota. Science, 304(5677), 1629–1633. 10.1126/science.1094875 15192218

[ece310667-bib-0068] Whitehead, S. R. , Bass, E. , Corrigan, A. , Kessler, A. , & Poveda, K. (2021). Interaction diversity explains the maintenance of phytochemical diversity. Ecology Letters, 24(6), 1205–1214. 10.1111/ele.13736 33783114

[ece310667-bib-0069] Wickham, H. (2016). ggplot2: Elegant graphics for data analysis. Springer‐Verlag. https://ggplot2.tidyverse.org

[ece310667-bib-0070] Wurst, S. , Wagenaar, R. , Biere, A. , & Van der Putten, W. H. (2010). Microorganisms and nematodes increase levels of secondary metabolites in roots and root exudates of *Plantago lanceolata* . Plant & Soil, 329(1/2), 117–126. 10.1007/s11104-009-0139-2

[ece310667-bib-0071] Zuppinger‐Dingley, D. , Flynn, D. F. B. , Brandl, H. , & Schmid, B. (2015). Selection in monoculture vs. mixture alters plant metabolic fingerprints. Journal of Plant Ecology, 8(5), 549–557. 10.1093/jpe/rtu043

